# Engaging Community–Academic Partnerships: A Case Study of the Influence of Social Drivers of Health on Equitable Transitions of Cancer Care in the United States

**DOI:** 10.3390/healthcare12020264

**Published:** 2024-01-19

**Authors:** Lailea Noel, Catherine Cubbin, Shetal Vohra-Gupta

**Affiliations:** Steve Hicks School of Social Work, University of Texas at Austin, Austin, TX 78712, USA; ccubbin@austin.utexas.edu (C.C.); sgupta@austin.utexas.edu (S.V.-G.)

**Keywords:** health equity, cancer care, community–academic partnerships, social drivers

## Abstract

Enduring community–academic partnerships are essential for expediting the successful dissemination and implementation of promising interventions and programs, particularly for complex public health issues such as cancer prevention and control. The purpose of this case study was to understand the combined voices of a diverse group of stakeholders to outline the essential factors needed to translate research into sustainable cancer care within communities. System dynamics group model building was used to develop system maps of the factors impacting equitable access to cancer care services among three stakeholder groups (Group A: survivors and family members, n = 20; Group B: providers and administrators in community agencies/organizations, n = 40; Group C: administrators from a cancer institute, academic universities, foundations, and healthcare facilities that coordinate care, n = 25) in central Texas, USA. The lead researcher identified factors involved in transitions of care and their linkages with each other. The analysis of this work displays these connections visually. These models represent the ripple effect of factors influencing the transition of care for stakeholders who are invested in cancer care outcomes. All three groups identified medical mistrust, a culturally sensitive and diverse provider workforce, and care coordination as three essential factors (i.e., themes). Group A also identified caregiver navigation. The groups varied in their emphasis on upstream vs. downstream social drivers of health, with Group B emphasizing the former and Group C emphasizing the latter. To achieve cancer care equity, all stakeholder groups agreed on the importance of addressing the impact of social drivers as critical gaps. Eliminating or reducing these impacts allows each stakeholder group to work more efficiently and effectively to improve cancer care for patients.

## 1. Introduction

While increased attention has been paid to cancer disparities and their influence on patient outcomes, interventions to eliminate disparities have been largely limited in reach and sustainability [1]. Historically, it was not common practice to tailor interventions to marginalized populations, including those in geographically isolated communities, whose problems they are meant to address [2]. As a result, more community–academic partnerships have emerged in the past decade to improve local health concerns and reduce health disparities [3]. It is becoming clear that these types of partnerships and coalitions are necessary to develop lasting health promotion and wellness across communities because no single provider institution has the resources, access, and relationships to address the vast range of social drivers of health in geographically isolated communities [4,5]. Public health challenges, such as cancer, have an expansive set of social circumstances that impact the cause and course of the disease [6]. Consequently, they require a multifaceted approach to address the complexities of implementing treatment practices [7,8].

Academic research and clinical practice knowledge often fail to translate from university-based development to real-world application. Alternatively, community organizations often lack investment in academic research, stating different needs than those addressed by the researchers [3,9]. The knowledge gap between research and community practice is evident. Yet, little success has been reported in the literature on how to close this gap. Most of the focus of the implementation of science in health has been on what works on paper rather than what works in practice for a healthcare center based on the community they serve [10,11,12]. There is even less research on including the community served in the process [13,14,15]. Thus, developing enduring community–academic partnerships is essential for successfully disseminating and implementing promising interventions and programs, particularly for complex public health issues such as cancer prevention and control. Moreover, involving community partners has improved communication and trust, produced valuable innovation, and decreased the marginalization of communities that have yet to benefit from research participation [4,16,17]. Understanding how best to incorporate collaboration between healthcare systems and communities can expedite the translation of research into practice and allow more evidence-based solutions to be implemented into practice [6].

Although the importance of community–academic partnerships is apparent, and there has been a recent push by funding organizations (both national and local) to encourage community-engaged research, a successful and sustainable partnership requires a systematic strategy and long-term commitment that exceed a simple community outreach strategy [3,8,16]. The partnership must include multiple stakeholders, including academics, practitioners, patients, and community members, committed to creating an environment of co-learning and capacity-building in which findings and knowledge benefit all members. This paper illuminates the findings from one such collaborative case study. This case study is a project that combined stakeholders from (1) those affected by cancer (survivors and family members), (2) community agencies/organizations providing services to cancer survivors, and (3) administrators from cancer institutes, academic universities, foundations, and healthcare facilities that coordinate care for cancer survivors. The results from this case study, the combined voices of this diverse group of stakeholders, outline the essential factors needed to translate research into sustainable practice within communities.

## 2. Materials and Methods

### 2.1. A Case Study Approach

This study utilized the system dynamics group model building (GMB) case study approach to develop system maps of the factors impacting equitable access to cancer care services [18]. A system is a set of factors converging to set the environment for a social phenomenon (i.e., the equitable transition of cancer care within marginalized communities). The resulting map is a visualization of these factors and how they are connected. Visually, these connections appear as a spider web, and like a spider web, pulling on one part of the net will produce a ripple effect throughout the entire web. The key to this research is to understand where to tug on the web to have the most significant impact on this system of factors leading to improved community health equity. No one understands the systems at play better than those who live and work within them. In GMB, informal causal maps and formal models developed by community stakeholders can be used to uncover and understand the sources of system behavior to then work within systems rather than against them to provide sustainable change [19].

### 2.2. Setting

The geographic area for this case study was a region in Texas referred to as Central Texas. In 2020, there were 787,375 cancer survivors in Texas [20]. As of 2020, Texas has 256 counties, and 27% (7.6 million people) are rural [20]. Many of these communities are geographically isolated from urban areas and experience high poverty rates and social drivers of health inequity, leading to poor health outcomes, lower mental health functioning, and limited access to resources and health care services [21]. Scientific evidence suggests that those living in higher areas of geographic isolation have later stages of cancer diagnosis, which leads to higher mortality and a lower quality of life for survivors. The twenty-county Central Texas region has 1.2 million people, and according to the age-adjusted invasive cancer incidence rates by county in Texas, 2016–2020, fourteen of those counties have higher rates of cancer mortality than the state’s rate [20]. For 2016–2020, the age-adjusted cancer death rate from prostate (157,785), breast (178,334), and colon and rectum (77,008) cancer accounted for 40% of the deaths from cancer in this region [20].

### 2.3. Methodological Process

For this case study, participants worked in small groups to document and draw relationships between all factors that impact the system of cancer service delivery, paying particular attention to those most impacted by the gaps in service delivery. This project combined the voices of three stakeholder groups. A GMB session, was held with each stakeholder group separately (n = 85), conducted between January 2019 and August 2019: (1) those affected by cancer (survivors and family members) (n = 20); (2) providers and administrators in community agencies/organizations providing services to cancer survivors (n = 40); and (3) administrators from a cancer institute, academic universities, foundations, and healthcare facilities that coordinate care for cancer survivors (n = 25). The participants of each stakeholder group were identified because they had a relationship with the stakeholder leadership of that group. Each stakeholder group’s leadership recruited the participating members for their respective groups and invited the lead researcher (L.N.) to facilitate the GMB session. For example, the Komen Foundation of Central Texas invited all participating non-profits who receive funding from their organization to support cancer patients. The session was designed to be the first step in reorganizing the organization’s future funding priorities.

The stakeholder groups hosted the GMB sessions, which took place within one of their facilities; for example, the session with providers and administrators in community agencies/organizations providing services to cancer survivors, was hosted by the Komen Foundation and took place at their office. The session with those affected by cancer was hosted by the young adults with a cancer advisory group from one of the providers, and the meeting took place at a public library after hours. The research team was invited into the project to facilitate the GMB process. One member of each stakeholder group served as a co-facilitator with the lead researcher (L.N.). Each GMB session took three hours.

The questions posed to the participants during the GMB sessions included the following:

What factors would need to be in the system if you could design one that allowed you to service the most marginalized communities in your region?
What are the gaps in service delivery for all communities, paying particular attention to the most marginalized groups?
What is needed within this system to provide ideal cancer care service delivery, including race, ethnicity, socioeconomic status, and those living in geographically isolated areas?

**Figure 1:**
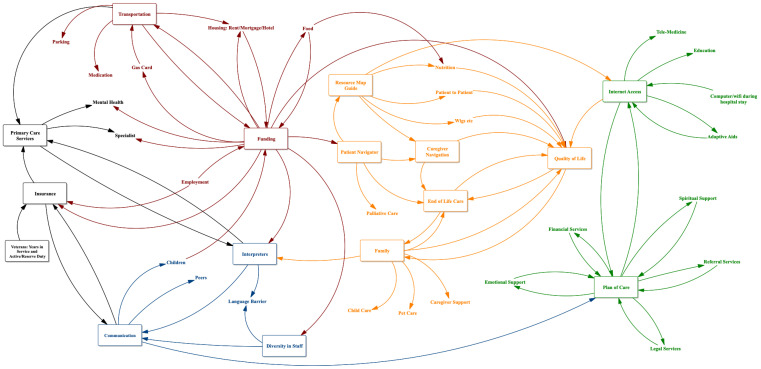
Model for Group A stakeholders: the ripple effect of factors influencing the transition of care for those affected by cancer (survivors and family members).

**Figure 2:**
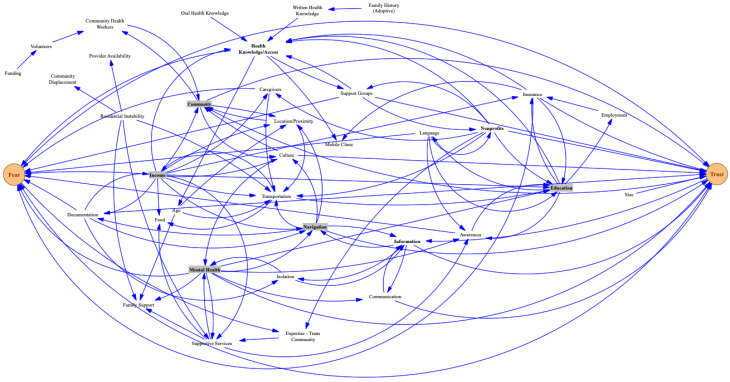
Model for Group B stakeholders: the ripple effect of factors influencing the transition of care for community agencies/organizations providing services to cancer survivors.

**Figure 3:**
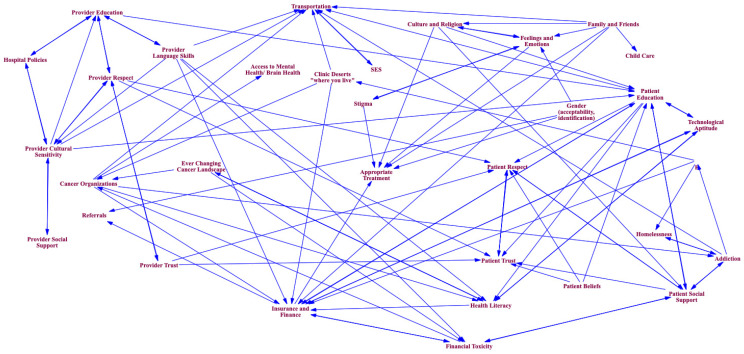
Model for Group C stakeholders: the ripple effect of factors influencing the transition of care for administrators from cancer institutes, academic universities, foundations, and healthcare facilities that coordinate care for cancer survivors.

During the GMB session, the participants were divided into smaller groups to facilitate discussion on the questions posed above. They were then asked to draw system maps of factors impacting equitable access to cancer care. They also drew lines between key factors, indicating an association between the factors (see Figure 1, Figure 2 and Figure 3). For example, a lack of diversity in staff at provider institutions influences language barriers between providers and patients. Yet, the presence of interpreters can offset this barrier, and all three factors (diversity of staff, language barriers, and interpreters) influence communication between provider and patient (Figure 1). The lead researcher (L.N.) used formalized and previously tested scripts to facilitate this process during each GMB session [18]. The sessions were audio-recorded.

The lead researcher analyzed each group’s discussion transcripts and drawings. The lead researcher looked for discussions including identifiable factors involved in transitions of care and their linkages with each other. The resulting conversation was a verbal description of something akin to a dream catcher. Tugging on one part of the web will produce a ripple effect on the other parts of the web. The analysis of this work was to display these connections visually. For example, an important part of transitions of care is access to information on one’s diagnosis. However, equitable access to health information is influenced by access to the internet, which is influenced by the availability of internet bandwidth in one’s community. The results were then entered into Vensim PLE 4.0, a software program for consolidating the information into a “causal map,” or a visualization of all the system factors shown, and connecting lines documenting the relationships between the factors for each stakeholder group [18].

A second modeling session was held with each stakeholder group to report back their generated system model in the participants’ words to validate the information. The investigators ensured that no words or concepts were added to the resulting models if the stakeholders did not provide them. This study summarized the narrative as it was reported by the participants to remain true to the qualitative voice of the community. This session lasted for 90 min and included the same participants present during the first session.

The final three stakeholder models were combined into one system causal map by the lead researcher (L.N.) to represent a global view of the (ideal) cancer care system as determined by the stakeholder groups. A combined meeting of participants from all three stakeholder groups was held. This town hall meeting was held to report back on the combined model and solicit the next steps of an implementation plan from the combined community-engaged group of stakeholders. This meeting took 90 min and was facilitated by the lead researcher (L.N.).

The accredited University Institutional Review Board of the research team was consulted, and it was determined that the project did not meet the definition of human subjects research because it was a quality improvement project.

## 3. Results

See Figure 1, Figure 2 and Figure 3 for the resulting models. These models represent the ripple effect of factors influencing the transition of care for stakeholders who are invested in cancer care outcomes. The key themes of these group conversations with stakeholders revealed a renewed interest in understanding the psychosocial factors influencing medical mistrust. Participants mentioned a need for a more diverse provider workforce, including increased mental health providers and more focus on cultural sensitivity within training programs. These conversations with stakeholder groups also highlighted the difficulty of coordinating care in an environment that includes an ever-changing cancer landscape coupled with residential instability and the displacement of communities most impacted by disparities. This section will outline these results. The stakeholder groups will be referred to as Group A (those affected by cancer (survivors and family members)), Group B (providers and administrators in community agencies/organizations providing services to cancer survivors), and Group C (administrators from a cancer institute, academic universities, foundations, and healthcare facilities that coordinate care for cancer survivors).

### 3.1. Medical Mistrust

Medical mistrust is an issue that has been extensively written about in the literature; however, a clear implementation path from research to practice has yet to be outlined. The results from this study begin to illuminate the elements needed in this pathway. All three stakeholder groups mentioned critical elements of medical mistrust. Group C (Figure 3) highlighted the importance of provider trust in and respect for patients and patient trust in and respect for providers as critical elements in equitable care. As a group of providers, their perspective was that bias exists on both sides. From their perspective, system factors influencing medical mistrust included provider education and cultural sensitivity, hospital policies, provider language skills, and the presence of interpreters. Their views of the patient perspective influencing medical mistrust included patient beliefs, health literacy, patient education, and the patient social support system. Group B explained that they define their roles and responsibilities as non-profit providers and administrators embedded in communities as moving patients and survivors from fear to trust. Their model (Figure 2) is a dynamic system of elements facilitating an environment of medical trust. Such elements include equipping communities with information that increases awareness and health literacy, access to mental health providers and other supportive services, and providing navigation services such as transportation and mobile clinics. These elements and others (see Figure 2) provide actionable items, increasing medical trust. Finally, when discussing medical trust with those affected by cancer (Group A), they responded that the resulting model is what trust looks like. Including the following elements (Figure 1) ensures an environment where they are seen and heard by providers: including interpreters and patient care services such as mental health and other specialists; improving patient navigation to include the navigation for the caregivers; providing a cafeteria plan of care to include emotional support, financial services, spiritual support, referral services, and legal services as needed. All of these elements, taken together, provide the guideline for a sustainable implementation plan for addressing medical mistrust within communities.

### 3.2. Culturally Sensitive and Diverse Provider Workforce

A culturally sensitive and diverse provider workforce is another vital area impacting equitable transitions in cancer care delivery. Each of the three stakeholder groups mentioned the importance of this issue. Group A (those affected by cancer) outlined a workforce that is not only diverse ethnically but also by discipline (see Figure 1). They want to see a workforce that includes more language interpreters, mental health providers, and specialists. Communication and access to a diverse workforce are essential to connecting and communicating with providers. They often feel more isolated because of a lack of these services. As an example, a parent of a child with cancer described a circumstance where there was an interpreter on duty at the hospital only for part of the day. On many occasions, the medical team would come to speak with them, and there would not be anyone to help bridge the language barrier. Group B (providers and administrators in community agencies/organizations) reported the importance of hiring community health workers and community navigators from the communities served to serve on clinical teams to address cultural sensitivity within clinical encounters. Including community health workers who represent their communities also diversifies the workforce. Group C (administrators coordinating care for cancer patients and survivors) spoke of reviewing and updating policies that impact hiring practices. A more diverse workforce will lead to more sustainable and equitable practice and policy implementation.

### 3.3. Coordination of Care within a Dynamic Community Landscape

A third category of findings influencing the implementation of equitable transitions of care within communities most impacted by social drivers of health involves the coordination of care. Group C (administrators coordinating care for cancer patients and survivors) described a system where therapies and protocols for treating illness are improved, and new ones are developed rapidly. Introducing information about new therapies and new public health messaging into marginalized and isolated communities takes a long time. This gap introduces a disparity in treatment uptake and increases medical mistrust. The providers and administrators in community agencies/organizations embedded within communities (Group B) described an ever-changing landscape due to gentrification. In many marginalized communities, gentrification has led to the displacement of those most impacted by social drivers of health. In the area covered for this study, marginalized groups have been displaced outside of the urban county and into a more isolated and rural county. However, the geographic boundary for the non-profits providing supportive services for this population has remained unchanged within the urban county. Group A (those affected by cancer) reported being caught between a rapidly advancing scientific community and their communities’ increasing geographic isolation and, in some cases, social isolation. Knowing where to go for information and how to access supportive services is challenging. Gaining a better understanding of these factors will improve the implementation of policies affecting the transition of cancer care services.

While the resulting causal system maps from all three groups pointed to the importance of the above three themes, some factors were important to specific stakeholder group maps.

### 3.4. Caregiver Navigation

Group A (those affected by cancer) highlighted the importance of patient navigation for caregivers. In many cases, whether it is parents of children and teens with cancer, family, and friends of rural cancer patients, or where English is not the first language for the cancer patients, the caregiver is a major partner in the healthcare journey of the cancer patient. In these examples, the caregiver interprets results and treatment plans, assists the cancer patient in connecting with care, and identifies sources of information and resources the cancer patient needs. Group A would like to see more support in the system for this group (see Figure 1). One new source of support would be a caregiver navigator, not just a patient navigator. In the same way that patient navigation addresses social drivers of health that lead to barriers to care, a caregiver navigator would work in partnership with the caregiver to decrease barriers to care for the patient. For example, a child with cancer needs radiation therapy, but English is not the first language of the parents, and the information on scheduling the appointment is confusing to the parents. As a result, the parents have chosen a relative to partner in their child’s health care. This caregiver has the information and requires assistance navigating the healthcare system. A caregiver navigator would be helpful. Some medical programs will pay for a patient navigator, but funding for a caregiver navigation program is scarce.

This group also discussed the inclusion of a resource map guide for caregivers. A resource map guide would include who to contact for certain questions and where to go if the family requires medical, social, or psychosocial assistance. This resource map guide would include information on tangible items such as wigs, patient-to-patient support groups, and medical groups that provide palliative care. Group A envisioned this as a road map for caregivers in partnership with those affected by cancer. This resource and caregiver navigators are essential to implementing equitable care transitions for Group A.

### 3.5. Upstream versus Downstream Factors

There are different ways to view the presence of social drivers of health within a system of care delivery. One could use a downstream lens (individual-level factors). Or one could view the system through an upstream lens (social conditions and policies, neighborhood conditions, and policies and procedures within provider organizations). It is worth noting that the system map for Group B (providers and administrators in community agencies/organizations) and Group C (administrators coordinating care for cancer patients and survivors) differ on the primary lens choice to view the factors required for equitable care transitions. Group C focused more on downstream factors such as the patient’s insurance status, health literacy and education, patient–provider respect and trust, and the provider’s understanding of the patient’s cultural beliefs. There was very little focus on upstream factors. Group B, on the other hand, focused almost exclusively on upstream factors. These factors included community displacement, community location, proximity to clinics, social services, community geographic isolation, and the availability of transportation. Including these perspectives in a single causal map will improve the implementation of care transition.

### 3.6. Town Hall Meeting

During the town hall meeting, the final full system map results were presented to the combined stakeholder group. Each group was given a causal model from a different group and asked to discuss it in small groups. One of the outcomes of this meeting was the new awareness of a different perspective on what is important to ensure equitable care transitions. Each group commented that they never thought of some of the factors presented by the other groups. This awareness of other ways to view the care delivery system was important to the strategic planning process. As a group, they also prioritized the factors in the combined model to decide where to begin to intervene.

## 4. Discussion

The United States Department of Health and Human Services Healthy People 2020 outlines the five domains of social determinants of health: Economic Stability, Education Access and Quality, Health Care Access and Quality, Neighborhood and Built Environment, and Social and Community Context (https://health.gov/healthypeople/priority-areas/social-determinants-health (accessed on 30 November 2023)). The results from this case study support that not only is understanding each of these domains essential to implementing a plan for equitable transitions of care for marginalized communities, but also by engaging a diverse group of stakeholders in the process and including their combined voices in creating solutions, we can improve sustainability and move closer to translating research into practice within communities.

It is well documented in the literature that social drivers of health influence health outcomes. However, more research should focus on the influence of these social drivers on implementing equitable healthcare service delivery. As a result, more community–academic partnerships have emerged in the past decade to improve local health concerns and transitions in care service delivery. It is becoming clear that these partnerships and coalitions are necessary to develop lasting health promotion and wellness across communities. This case study examined what factors are needed within these pathways to create a sustainable implementation plan to address equitable transitions in healthcare service delivery.

Including all stakeholders in the implementation process from the start of planning, including developing the aim and scope of the process, achieves several goals. This manuscript will describe three: (1) members of marginalized groups who are most impacted by social drivers of health receive the care packaged in a way that is most accessible to them; (2) communication between stakeholder groups is improved, thus expediting the delivery of information and resources within geographically isolated communities; and (3) the capacity to implement evidence-based practice is increased.

Including all stakeholders in the implementation process from the start of planning ensures that members of marginalized groups who are most impacted by social drivers of health receive the care packaged in a way that is most accessible to them. An example is tailoring a colon cancer screening to fit a community’s cultural and social norms. Hispanic men have high rates of colon cancer and yet the lowest rate of colon cancer screening [22]. The system needed to improve the screening program implementation for this group will look different than other groups. Including a diverse group of stakeholders in the process identifies factors required in an implementation plan from various viewpoints, including those most impacted by the inequity in the process.

In addition, including all stakeholders in the implementation process from the start of planning improves communication between stakeholder groups, thus expediting the delivery of information and resources within geographically isolated communities. For example, often, healthcare programs have developed new therapies or the availability of clinical trials, and yet the dissemination of this information to all communities is a challenge. Including a diverse group of stakeholders in the process also creates a diverse group of champions who can assist with translating new therapies into communities. These champions have already earned their constituents’ trust and know where, when, and how to disseminate the information. Similar to the spokes of a wheel, these champions can deliver information on new therapies or clinical trials to diverse groups through trusted vehicles.

In addition, as outlined in Section 3, each stakeholder group approaches the implementation of therapies from their vantage point. For the administrators coordinating care for cancer patients and survivors, the focus was on downstream factors whereas the providers and administrators in community agencies/organizations embedded within communities focused almost exclusively on upstream factors. This makes sense based on the daily operations of their roles and responsibilities. However, when rolling out the communication plan for a new therapy or clinical trial in geographically isolated communities, it is important to include many perspectives, both upstream and downstream, to address more of the patient experience. Approaching this effort by including a diverse group of stakeholders at the table lends itself to this goal.

Finally, partnering with different stakeholders builds the capacity to implement evidence-based practice. Providers of cancer care need sufficient resources, tools, and individuals to move science to practice. An increase in capacity requires the support of leadership and funding within these organizations. Most organizations require proof of best practices before supporting the implementation of the practice. Providers can document the success of equitable implementation by including a diverse group of stakeholders in the implementation process from the start. Providers strengthen capacity by engaging various stakeholders in evaluating and improving evidence-based practice training, assessment, and efficacy.

### 4.1. Policy Implications

In response to the critical findings presented in this study, policy implications and recommendations are proposed to guide further the development and implementation of interventions to promote equitable cancer care for marginalized communities. Policy implications on cross-sector collaboration allow the health sector to create a more holistic approach to cancer care. First, given the significant impact of the role of community–academic partnerships as evidenced in the GMB methodology, an increase in funding from national and local healthcare foundations and other grant-giving institutions will foster long-standing collaborative initiatives. Second, administrators and leadership within cancer care centers or other healthcare centers should adopt an inclusive approach of involving diverse stakeholders such as patients and their families, survivors, community organizations, and healthcare providers in the policy-making process related to cancer care and health equity. The importance of including all voices or multiple perspectives in the policy-making process allows for culturally sensitive and sustainable policies that work to build trust and increase buy-in for engagement in cancer-related treatment. Third, the incorporation of GMB into strategic planning for healthcare institutions, academic research units, and grant-giving institutions is a valuable tool for gaining a comprehensive understanding of gaps in cancer care, especially for marginalized populations. This method centers on community voices and the voices of healthcare providers and community organization leaders, all necessary to build respect, trust, and health equity within the cancer care space.

Through this work, the call to action includes policy recommendations within healthcare organizations and institutions. First, in addressing the critical issue of medical mistrust, developing and supporting culturally sensitive training, including creating an environment of cultural humility, is recommended for cancer care centers and other relevant healthcare settings. In addition, 24/7 availability of language services for patients, families, and caregivers who are navigating cancer care is needed to build trust. The findings also point to policies that support recruitment, training, and retention initiatives to build a diverse workforce that can effectively build trust and address the needs of marginalized communities. Another policy recommendation includes allocating resources for caregiver navigation programs and resource map guides. The policy recommendations presented based on the findings of this study aim to improve the quality of life for patients, their families, and survivors. The GMB, based on the voice of this stakeholder group (Group A), recognizes that quality of life is impacted for the better when policies like the ones mentioned here are developed and implemented.

Policy implications for impacting social drivers of health for this community involve developing policies and practices that account for residential instability and displacement, transportation needs, food and nutrition access, mental health services, and better culturally responsive communication around health literacy and health knowledge. While these implications are not new to the literature, the findings from this study highlight the overlap of all stakeholder groups, mentioning the importance of addressing social drivers as critical gaps. Eliminating or reducing these social barriers allows each stakeholder group to work more efficiently and effectively to improve cancer care for patients.

### 4.2. Strengths and Limitations

A strength of this research is that it explores services and resources needed to connect marginalized communities with cancer care and identifies the policy changes required to improve trust, timely access to care, and supportive services. Partnerships between cancer centers, health departments, social work, and communities deliver sustainable tools with important implications for policy change and the increasing utilization of health services by those most impacted by health inequity. In addition, the GMB methodology utilized here is transferrable to other communities. That being said, several limitations exist within this work. First, while this study provided valuable insights into the factors that influence the equitable transition of cancer care in Central Texas, it cannot be generalized to other regions or communities without caution. Second, while this methodology is valuable for understanding complex systems, it may introduce subjectivity in the interpretation of results from the different stakeholder groups due to the reliance on self-reported data during the GMB sessions. Along these lines, the potential for bias in the facilitation process due to the researcher’s lived experiences and perspectives should be acknowledged. Third, future research should include more stakeholder groups from other regions as well as different stakeholder groups such as policymakers, insurance representatives, and individuals from different cultural backgrounds to offer more comprehensive system maps.

## 5. Conclusions

The key themes of these group conversations with stakeholders revealed an interest in understanding factors influencing medical mistrust, a critical need for a more diverse provider workforce with training on cultural sensitivity, and the difficulty of coordinating care in an environment that includes an ever-changing cancer landscape coupled with residential instability and the displacement of communities. Caregiver navigation and an upstream vs. downstream lens were also apparent for specific stakeholder groups. Future research should investigate whether and how stakeholders may have changed their views after experiencing the COVID-19 pandemic. By engaging a diverse group of stakeholders in the process and including their combined voices in creating solutions, we can move closer to translating research into sustainable practice within communities.

## Data Availability

The data for this study are not publicly available due to the privacy statement agreed upon in the informed consent with the participating stakeholder organizations.

## References

[1] Gehlert S., Coleman R. (2010). Using community-based participatory research to ameliorate cancer disparities. Health Soc. Work.

[2] Jenkins C., Pope C., Magwood G., Vandemark L., Thomas V., Hill K., Linnen F., Beck L.S., Zapka J. (2010). Expanding the chronic care framework to improve diabetes management: The REACH case study. Prog. Community Health Partnersh. Res. Educ. Action.

[3] Drahota A., Meza R.D., Brikho B., Naaf M., Estabillo J.A., Gomez E.D., Vejnoska S.F., Dufek S., Stahmer A.C., Aarons G.A. (2016). Community-academic partnerships: A systematic review of the state of the literature and recommendations for future research. Milbank Q..

[4] Wallerstein N.B., Duran B. (2006). Using community-based participatory research to address health disparities. Health Promot. Pract..

[5] Baker E.A., Homan S., Schonhoff S.R., Kreuter M. (1999). Principles of practice for academic/practice/community research partnerships. Am. J. Prev. Med..

[6] Noel L., Chen Q., Petruzzi L.J., Phillips F., Garay R., Valdez C., Aranda M.P., Jones B. (2022). Interprofessional collaboration between social workers and community health workers to address health and mental health in the United States: A systematised review. Health Soc. Care Community.

[7] Wolff M., Maurana C.A. (2001). Building effective community—Academic partnerships to improve health: A qualitative study of perspectives from communities. Acad. Med..

[8] Alexander J.A., Weiner B.J., Metzger M.E., Shortell S.M., Bazzoli G.J., Hasnain-Wynia R., Sofaer S., Conrad D.A. (2003). Sustainability of collaborative capacity in community health partnerships. Med. Care Res. Rev..

[9] Ross L.F., Loup A., Nelson R.M., Botkin J.R., Kost R., Smith G.R., Gehlert S. (2010). The challenges of collaboration for academic and community partners in a research partnership: Points to consider. J. Empir. Res. Hum. Res. Ethics.

[10] Farley-Ripple E., May H., Karpyn A., Tilley K., McDonough K. (2018). Rethinking connections between research and practice in education: A conceptual framework. Educ. Res..

[11] Fixsen D., Blasé K.A., Metz A., Van Dyke M., Wright J. (2015). Implementation science. International Encyclopedia of the Social & Behavioral Sciences.

[12] Nelson J., Campbell C. (2017). Evidence-informed practice in education: Meanings and applications. Educ. Res..

[13] Anderson L.M., Adeney K.L., Shinn C., Safranek S., Buckner-Brown J., Krause L.K. (2015). Community coalition-driven interventions to reduce health disparities among racial and ethnic minority populations. Cochrane Database Syst. Rev..

[14] South J., Phillips G. (2014). Evaluating community engagement as part of the public health system. J. Epidemiol. Community Health.

[15] Schensul J.J., Trickett E. (2009). Introduction to multi-level community based culturally situated interventions. Am. J. Community Psychol..

[16] Plowfield L.A., Wheeler E.C., Raymond J.E. (2005). Time, tact, talent, and trust: Essential ingredients of effective academic-community partnerships. Nurs. Educ. Perspect..

[17] Greenhalgh T., Jackson C., Shaw S., Janamian T. (2016). Achieving research impact through co-creation in community-based health services: Literature review and case study. Milbank Q..

[18] Hovmand P.S. (2014). Community Based System Dynamics.

[19] Stroh D.P. (2015). Systems Thinking for Social Change: A Practical Guide to Solving Complex Problems, Avoiding Unintended Consequences, and Achieving Lasting Results.

[20] Texas Cancer Registry (2023). Age-Adjusted Invasive Cancer Incidence Rates by County in Texas, 2016–2020. Cancer Incidence File. http://cancer-rates.info/tx/.

[21] Gamm L., Hutchison L. (2003). Public health rural health priorities in America: Where you stand depends on where you sit. J. Rural Health.

[22] Spencer J.C., Noel L., Shokar N.K., Pignone M.P. (2023). Understanding the role of access in Hispanic cancer screening disparities. Cancer.

